# Intriguing correlations between leaf architecture and intrinsic water‐use efficiency enable selective breeding to mitigate climate challenges

**DOI:** 10.1111/pce.14305

**Published:** 2022-03-23

**Authors:** Paul Christiaan Struik, Steven Michiel Driever

**Affiliations:** ^1^ Centre for Crop Systems Analysis Wageningen University and Research Wageningen The Netherlands

Growing crops in a changing climate requires drought tolerance and strong recovery ability from drought spells. Scientists, growers and policymakers are faced with the challenge to create crops and cropping conditions that allow more crop per drop, whether that drop comes from rainfall or irrigation water. For crop scientists, this translates into creating crops with a higher water‐use efficiency of crop productivity (more biomass or yield per unit of water received either by rainfall or irrigation) or into a higher transpiration efficiency (more dry matter or yield produced per amount of water transpired by the crop) (Leakey et al., 2019). For plant scientists, this translates into more net photosynthesis per unit of water transpired at leaf level. This is called intrinsic water‐use efficiency (*iWUE* in μmol CO_2_ (mol H_2_O)^−1^), commonly quantified as the net photosynthesis (*A*_n_ in μmol CO_2_ m^−2^ s^−1^) divided by stomatal conductance for water vapour (*g*_sw_ in mol H_2_O m^−2^ s^−1^) (Medlyn et al., 2017). Note that *iWUE* only includes the water loss through the stomata and not the water loss through the epidermis of the leaf. *iWUE* is affected by climate change through the combined action of a multitude of factors: elevated CO_2_ concentrations in the ambient air, elevated temperature, increases in variation in soil moisture and precipitation (e.g., delays of monsoon, variable rainfall, increasing frequency and duration of drought spells), increases in variation in relative humidity (Hatfield & Dold, 2019) and incoming radiation and clarity of the sky (Gao et al., 2018). In particular, fluctuating light plays an important role in *iWUE* at leaf level because of the relatively slow response of stomata to light in comparison with photosynthetic processes (Lawson & Vialet‐Chabrand, 2019).

## 1 TOWARDS AN INCREASE IN INTRINSIC WATER‐USE EFFICIENCY

One important way to produce more crop per drop is to make use of the interspecific and intraspecific variation in *iWUE*, either for C_3_ or for C_4_ crops. There is abundant variation in *iWUE* among (Yi et al., 2019) and within (Hatfield & Dold, 2019) C_3_ crop species. C_4_ crop species have a higher *iWUE* than C_3_ plants. The main reason for this difference between photosynthesis types is that the CO_2_ concentrating mechanism of C_4_ species allows them to operate efficiently at lower stomatal conductance (Leakey et al., 2019). Therefore, C_4_ crops could be very useful in sustainable food systems under future climates with increasing chances of drought spells, increasing temperatures associated with enhanced transpiration, and therefore increasing water needs. C_4_ crop species also demonstrate variation in *iWUE*, both between C_4_ grasses (Cano et al., 2019; Pathare et al., 2020) and within important C_4_ crop species (Cruz de Carvalho et al., 2011; Feldman et al., 2018; Li et al., 2017). The mechanisms behind that variation in C_4_ crops are less clear than for C_3_ crops.

*iWUE* is difficult to assess on large numbers of genotypes, is time‐consuming, and requires sophisticated equipment and advanced skills. Both components of *iWUE* (photosynthesis and stomatal conductance) are very sensitive to fluctuations in environmental factors, such as light, temperature, and relative humidity, although Gu et al. (2012) demonstrated that statistical or physiological modelling approaches might help to account for those fluctuations. Overall, this makes *iWUE* a much‐needed, but difficult trait to select for in breeding. There is an urgent need for robust *iWUE* selection methods that can unlock the inter‐ and intraspecific variation for breeding better crops.

## 2 INDIRECT SELECTION FOR ENHANCED INTRINSIC WATER‐USE EFFICIENCY

For C_3_ crops, it has been shown that indirect selection based on relations between leaf width (*LW*) or vein characteristics and *iWUE* might be helpful (e.g., Feldman et al., 2017). But does this also work for C_4_ crop species with their CO_2_ concentrating mechanism? Could *LW* be an indirect selection criterion for drought tolerance and high water‐use efficiency in C_4_ crops? Answers come from an intriguing paper by Pan et al. (2021) published in this issue of *Plant, Cell & Environment*.

Previously, Cano et al. (2019) demonstrated that *LW* correlates strongly with *g*_sw_ and *iWUE* across C_4_ grass species and that *iWUE* depends more on *g*_sw_ than on *A*_n_. Pathare et al. (2020) showed that vein density correlates negatively with *iWUE*, while Ogle (2003) showed that vein density positively correlates with a quantum yield of photosynthesis across C_4_ grasses. Pan et al. (2021) investigated whether such correlations between morphological/anatomical traits and *iWUE* can also be found within a C_4_ crop species and what the mechanisms are behind them in sorghum (*Sorghum bicolor* (L.) Moench.), a suitable crop for these research questions because of its huge variation in and high heritability of *LW*.

Pan et al. (2021) grew 48 sorghum genotypes with large variation in *LW* and different genetic backgrounds under rain‐fed conditions, to assess whether *LW* correlated with *iWUE* and what the mechanism behind such a relationship was. They carried out leaf gas exchange measurements on fully expanded, but young leaves during the morning, mid‐day, and afternoon and measured leaf morphological and anatomic traits, including stomata and vein traits. Particularly, they elegantly assessed the % stomatal aperture as the ratio of the operational stomatal pore area to the mean stomatal pore area when fully open. Vein traits measured included leaf vein density (also called total vein length per leaf area; *V*_D_), including both longitudinal and transversal veins, and interveinal distances. Finally, the authors estimated the leaf boundary layer conductance and the leaf energy balance.

**Figure 1:**
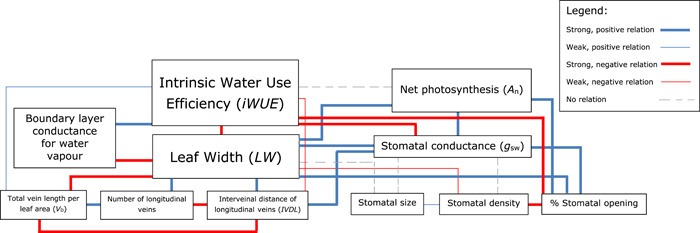
Relationships between anatomical, morphological and physical characteristics contributing to variation in leaf width and intrinsic water‐use efficiency (*iWUE*) in the C_4_ crop sorghum [Color figure can be viewed at wileyonlinelibrary.com]

**Figure 2:**
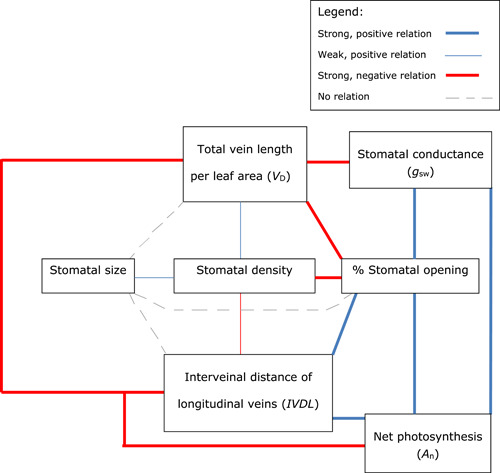
Detailed relationships between the vein characteristics total vein length per leaf area and interveinal distance between longitudinal veins and stomatal characteristics stomatal size, stomatal density and % stomatal opening in the C_4_ crop sorghum [Color figure can be viewed at wileyonlinelibrary.com]

*LW* varied 2.6‐fold among the 48 sorghum genotypes, while *g*_sw_ (3‐fold), *A*_n_ (2.4‐fold), and *iWUE* (1.6‐fold) also varied widely among genotypes and throughout the day. Figures 1 and 2 illustrate the correlations identified in the paper by Pan et al. *LW* correlated strongly (positively, linearly) with *g*_sw_ and *A*_n_, but negatively with *iWUE* (Figure 1). Interestingly, these linear correlations all disappeared in the afternoon. *iWUE* was mainly associated with changes in *g*_sw_ (negative correlation) rather than with changes in *A*_n_ (Figure 1). There was a negative correlation between *LW* and *V*_D_, a positive correlation between *LW* and interveinal distance of longitudinal veins (*IVDL*), and total number of longitudinal veins, a negative correlation between *LW* and stomatal density, and no correlation between *LW* and stomatal size. *V*_D_ was positively correlated with stomatal density, but negatively with *IVDL*, *g*_sw_, % stomatal opening and *A*_n_ (Figure 2). There were no correlations of *g*_sw_ or *A*_n_ with anatomical stomatal traits, strong, positive correlations of both *g*_sw_ and *A*_n_ with % stomatal opening (Figure 2), and a negative correlation between *iWUE* and % stomatal opening (Figure 1). Most interestingly, however, was the positive correlation between *LW* and % stomatal aperture during the morning and at midday (Figure 1), suggesting that, at least during part of the day, genotypes with wider leaves have their stomata more open than those with narrower leaves, resulting in a lower *iWUE* and indicating that % stomatal opening was a crucial factor in determining variation in *iWUE*.

*LW* was also associated with the boundary layer thickness and the associated boundary layer conductance for water vapour was strongly correlated with *iWUE* (Figure 1). Leaf energy balances at different levels of wind speed, various differences between leaf and air temperatures and during different parts of the day showed that *LW* correlated positively with *g*_sw_ because wider leaves had a wider stomatal opening, and this associated well with the energy balance of the leaves in the morning and at midday.

The lack of correlations during the afternoon was perhaps associated with the mild stress that occurred at that time of the day as the experiment was rainfed. This mild stress was visible by occasional leaf rolling, occurring earlier with narrower leaves, thus saving water.

## 3 SOME ASPECTS REQUIRING FURTHER RESEARCH

While the detailed analysis of the correlations between leaf anatomy and morphology with *iWUE* is intriguing, the analysis of the temporal dynamics is very insightful, the leaf energy balance provides additional triangulation and the paper contains an interesting discussion on scale issues in water‐use efficiency. Still, more quantitative research is required to scale up to canopy level (Table 1). Hammer et al. (2020) showed that genetic variation in crop level water‐use efficiency was rather small, although not insignificant, in contrast to the findings at leaf level reported by Pan et al. (2021). On the other hand, Condon et al. (2004) stated that *iWUE* does scale with crop water‐use efficiency under field conditions.

**Table 1:** Emerging research questions

**Scaling up research questions**:
Does *iWUE* scale up to crop *WUE*?
**Scaling down research questions**:
How does *LW* affect microanatomy and 3D pathways of water, CO_2_ and O_2_ inside leaves?
With low stomatal opening, how does epidermal conductance affect *iWUE*?
Are differential dynamics of stomatal behaviour and photosynthesis under fluctuating conditions also affected by *LW*?
**Specific questions on relation between vein structure and *iWUE* aspects**:
How does vein structure relate to stomatal density, size and behaviour?
How does vein structure relate to net photosynthesis?

Abbreviations: 3D, three‐dimensional; *iWUE*, intrinsic water‐use efficiency; *LW*, leaf width.

Scaling down, *LW* determines the three‐dimensional (3D) tissue arrangement of a leaf to a considerable extent, which is relevant for gas exchange. There is more research necessary on the 3D pathways of water vapour, CO_2_, and O_2_ inside the microanatomy of the leaves, including water loss through the epidermis (Table 1). Precise measurements of multidirectional movement of gases inside the leaves, combined with innovative approaches of measuring tissue geometries (Earles et al., 2019) and microscale modelling of gas exchange in C_4_ (Retta et al., 2022) are relevant to validate all kinds of traditional assumptions about the true conditions inside a leaf and thus mechanisms controlling *iWUE*. Recently, the conductance of the epidermis to water vapour was identified as a relevant factor when % opening of the stomata is very small and it, therefore, affects *iWUE* under such conditions (Márquez et al., 2021).

In the field, conditions are dynamic and can fluctuate at relatively small time steps. Relative response time of the various processes associated with *iWUE* then becomes relevant (Table 1). Stomatal opening requires energy which can come from photosynthesis or respiration. Therefore, there is a direct link between photosynthesis and stomatal opening (Vialet‐Chabrand et al., 2021). The light spectrum is important: blue light is more effective in opening the stomata than red light (Vialet‐Chabrand et al., 2021). At the same time, photosynthesis responds an order of magnitude faster to fluctuating light than stomata can (McAusland et al., 2016; Taylor and Long, 2017), even though Taylor and Long (2017) proved that fluctuating light also impedes photosynthesis. When environmental conditions are very dynamic *A*_n_ and *g*_sw_ may be temporally decoupled, causing a reduction in *iWUE*. This decoupling should ideally be prevented, as highlighted by for example, Lawson and Vialet‐Chabrand (2019), who demonstrated that ‘speedy stomata’ can enhance *iWUE*.

In short: *LW* is an intriguing and promising variable with important genetic variation and a large impact on many physiological aspects relevant for *iWUE* that deserve to be unravelled in more detail at different temporal and spatial scales for proper use in breeding for drought tolerance.

## CONFLICTS OF INTEREST

The authors declare that there are no conflicts of interest.
